# How can we begin to measure recovery?

**DOI:** 10.1186/1747-597X-5-31

**Published:** 2010-12-07

**Authors:** Karen Dodge, Barbara Krantz, Paul J Kenny

**Affiliations:** 1 Hanley Center, USA; University of Miami Miller School of Medicine, USA; Nova Southeastern University, USA; 2 Laboratory for Behavioral and Molecular Neuroscience, Department of Molecular Therapeutics, The Scripps Research Institute - Scripps Florida, USA

## Abstract

**Background:**

There is a lack of consensus in the addiction treatment literature regarding the definition of substance abuse "recovery".

**Methods:**

This study utilized a review of the literature together with a participatory research design to construct a conceptual model of recovery from the perspectives of addiction treatment professionals, those recovering from addictions, and researchers.

**Results:**

A multidimensional, comprehensive hypothetical model consisting of seven conceptual domains (physical, biomarker, psychological, psychiatric, chemical dependency, family/social, and spiritual) is presented. Each domain is operationally defined by identifying reliable and valid instruments that may be used to measure the domain. It is proposed that the conceptual model be tested using confirmatory factor analysis.

**Conclusions:**

If empirically supported, this conceptual model would validate the hypothesized multidimensional nature of recovery and provide a potential means for assessing recovery in future treatment outcome studies.

## Background

The field of addiction treatment lacks a universally accepted and unambiguously defined clinical definition of recovery. Although a single disciplinary group such as physicians may agree upon a definition, there is no such agreement among the broader field of treatment professionals, addiction researchers, program evaluators, and policymakers. Indeed, there is no comprehensive consensus of what a definition of "recovery" is even among those individuals who are themselves in recovery from substance use disorders [1,2].

There have been many efforts to define recovery from substance use disorders. Perhaps the most often used criteria for remission from substance use disorders by treatment professionals are those from the *Diagnostic and Statistical Manual of Mental Disorders *(*DSM*) [3]. An example of such a criterion is the sum of all years in which a diagnosis of alcohol use disorder was not present. However, this criterion indicates a remission from a clinical diagnosis, rather than a multidimensional perspective on recovery.

Perhaps the most comprehensive efforts to define "recovery" were those offered by leading investigators as part of a special issue on "recovery" published by the *Journal of Substance Abuse Treatment *(October 2007). Methodological approaches to developing a definition of recovery included a consensus panel, literature reviews, surveys of the general public and addiction treatment professionals, and opinions from persons themselves in "recovery".

The Betty Ford Consensus Panel [4] defined "recovery" as consisting of three parts: sobriety, personal health, and citizenship. Sobriety refers to abstinence from alcohol and all other non-prescribed drugs; personal health refers to improved quality of health; and citizenship refers to living with regard and respect for others. This conceptualization has come under criticism for several reasons, including the use of the construct of citizenship as a measure of chemical dependency recovery. The objection is that no other chronic illness is measured for recovery status on the construct of citizenship [5]. The Betty Ford panel proposed measuring recovery using the World Health Organization Quality of Life instrument. However, this approach has been critiqued on the ground that a measure developed for a general population may not be valid for the specific population of people in recovery [5]. Arndt & Taylor [5] view the Betty Ford conceptualization as an initial step in defining "recovery," rather than the pinnacle of a definition.

White [6] defined "recovery" as the experience (a process and sustained status) through which individuals, families and communities impacted by severe alcoholism and other drug problems utilize internal and external resources to voluntarily resolve these problems, heal the wounds inflicted by alcohol and other drug-related problems, and develop a healthy, meaningful and productive life. White [7] subsequently proposed outcome measures for these areas including measures of substance use, living environment, physical and emotional health, family relationships, citizenship, and quality of life.

Laudet [1] cites a survey of members of the public regarding their view of recovery (Peter D. Hart Research Associates, 2004). This survey indicated that 62% reported that "in recovery from addiction to alcohol or other drugs, the one addicted is trying to stop using". Only 22% of respondents reported that "the one in recovery is free from the disease of addiction and no longer uses alcohol or illicit drugs". Further, 80% of respondents expressed that total abstinence was their "recovery" goal and over 80% reported that "recovery" is a process and not a finite achievement. Apart from the public's perceptions, Laudet [1] also conducted a review of articles on recovery and concluded that most researchers operationally define recovery in terms of substance use and more often as abstinence status. Some of the terms used interchangeably were *remission, resolution, abstinence, and recovery*. In addition, words to represent the act of changing the substance using behaviors were *quit, overcome, and recover*. In these contexts, "recovery" is defined as "overcoming both physical and psychological dependence to psychoactive drugs while making a commitment to society." This description implies domains of recovery that encompass drug abstinence, personal wellbeing, and re-integration into society.

Galanter [8] suggested a model of "recovery" from addiction that is attuned with the spiritual framework supported by Alcoholics Anonymous. This aspect of recovery is based on the substance-using individuals' own perspectives. These experiences are not observable; rather, they are self-reported through the persons' interpretations. This is an important domain of "recovery" and is reminiscent of the spiritual orientation of Alcoholics Anonymous.

Finally, McLellan, Chalk, & Bartlett [9] present "recovery" in terms of outcomes, performance, and quality. Calls for accountability within the addiction treatment field have inspired these authors to build a set of treatment quality, performance and outcome indicators. They suggest that outcomes of any treatment are the changes in clients' symptoms, behaviors, and functioning that can be attributed to the treatment. Because clients present with multiple problems, outcome evaluations of chemical dependency treatment have measured more than one outcome variable. Outcome measures are generally grouped together by the domain of functioning that they represent. When clients experience abstinence or a substantial reduction in use of drugs/alcohol as well as improvement in functioning in other domains (e.g., family, social, education, financial, etc.) this can be called "recovery." The three variables that are most frequently presented as "recovery" domains are substance use, employment/self-support, and criminal activity. The Substance Abuse and Mental Health Services Administration [10] measures substance abuse "recovery" by adjoining physical health, mental health, family and social relations, stability in housing, perception of care, access, and retention domains. According to SAMSHA, improvements in three of the seven functional domains plus abstinence are considered indicative of "recovery".

As can be seen from the above, the definition and very concept of recovery is unclear, although a convergence of ideas is beginning to emerge. It might be fundamental to consider the foregoing efforts, which represent collective federal agency/consensus panel/empirical definitions of "recovery" as a starting point meant to be reviewed, revised, expanded upon, revisited, and updated. Therefore, the purpose of this article is to move toward an abstinence-based model of recovery by using existing models and developing upon them. This article is theoretical in nature and does not present quantitative analyses. Instead it presents the results of a small qualitative effort meant to create a theoretical foundation for future research.

## Methods

This study used a participatory approach to explore "recovery" from the perspectives of addiction treatment professionals. It used inductive content analysis approaches to identify domains of recovery that could be modeled for further testing.

### Setting

The study was conducted at the Hanley Center, a private, 82-bed residential substance abuse treatment facility located in South Florida. The Hanley center serves a primarily white, private-pay clientele. The facility has separate treatment centers for men, women, and older adults. Comprehensive assessment and services are provided based on an eclectic model including medical, 12-step, cognitive/behavioral, and spiritual approaches delivered in individual, family, and small group modalities.

### Participants

There were eleven participants. Eight of these were managers of their respective disciplines at the Hanley Center, and three were researchers. The following disciplines or treatment perspectives were represented among the eight managers: medicine (physician and nurse); psychiatry; psychology; clinical therapy; social/emotional wellness; family/community; and spirituality. The multidisciplinary approach was used in order to obtain a comprehensive perspective on recovery. The researchers, who had expertise in addiction, research methodology, and neuroscience, lent their empirically-centered guidance to the process. Some of these participants were in recovery themselves. The participants included both men and women, all had a master's or doctorate degree in their discipline, and most had worked in the field of substance abuse for more than ten years.

### Procedures

The participants convened in group discussions at the Hanley Center over the period of one year to review literature, review the instruments used to collect data from Hanley clients, discuss practice observations and experiences, and derive findings in a prioritization process to define "recovery" and select measures to indicate the attainment of "recovery." The researchers solicited and encouraged the managers/practitioners to inductively construct domains from the ground up to represent dimensions of "recovery." Their responses were then merged with the existing literature to determine overlaps of concepts and reconcile differences in order to formulate a collective operational definition of "recovery" and select instruments that measure it.

## Results

The literature review and group discussion process led to the conclusion that a single industry-wide definition of "recovery" did not exist and that the multiple definitions that did exist were usually subjective and open for interpretation and often appeared more ideological than scientific. As a result of this process and conclusions, the participants inductively constructed seven domains that represent aspects, and are sensitive to status, of "recovery": physical; bio-marker; chemical dependency; psychological; psychiatric; family/social; and spiritual. This conceptual model is summarized in Table 1. For each conceptual domain, potential operational measures are proposed. Some of these measures were already in use at the Hanley Center at the time of the group discussions, but others were not and were selected based on the literature review.

**Table 1:** Conceptual Model of Substance Abuse Recovery

Conceptual Domain	Potential Operational Measures
Physical	History and Physical
	Urine Chemistry Panel
	Brain Imaging
	ASI Medical Subscale
	
Bio-Marker	Neuropeptides: Orexin, Oxytocin
	Cortisol
	
Chemical Dependency	ASI Alcohol/Drug Use Subscales
	Urine and Breath Screening
	
Psychological	MMSE or TONI-3
	Shipley
	
Psychiatric	DSM-IV-TR Axis I
	ASI Psychiatric Health Subscale
	
Family/Social	ASI: Employment/Self-Support, Family Relations, Illegal Activity Subscales
	MMPI-2: Work Interference, Family Problems, Antisocial Practices Scales
	
Spiritual	Spirituality Self-Rating Scale
	ASI-JCAHO Spirituality Scale

The model is holistic in that all the domains are hypothesized to be correlated with each other and all are considered important to recovery. However, it should be noted that not all domains will be relevant to all clients. For example, some addiction clients have co-occurring physical and psychological impairments wherein improvement may not be expected, and thus these domains would not be relevant elements of recovery for these individuals.

### Definitions and Measurement of Domains

Each of the domains is defined below and the instruments that may be used to measure it are identified. All identified instruments have established reliability and validity.

#### Physical Domain

Recovery is indicated in part by improvement in overall physical health. This may be measured by a history and physical examination, blood panels, brain imaging, and self-report. History, physical exam, and blood panels may be used to measure general signs, symptoms, and chemistries indicative of health and illness. Brain imaging using scans such as SPECT, PET, MRI, and functional MRI may be used to assess the effects of abused substances on the brain. Finally, the self-report medical health subscale of the Addiction Severity Index (ASI) [11] may be used to measure the physical domain.

#### Bio-Marker Domain

Kraemer, Schultz, & Arndt [12] have defined a biomarker as a measurable characteristic of living tissues that indicates whether a specific event or process of medical interest has occurred, is occurring, or is likely to occur. Frank & Hargreaves [13] define a biomarker as a characteristic that is objectively measured and evaluated as an indicator of normal biological processes, pathogenic processes, or pharmacological responses to a therapeutic intervention. The common theme captured in the definitions above is that biomarkers can provide readily accessible information regarding an individual's disease state.

We propose that relevant biomarkers for measuring recovery include neuropeptides and hormones. Specifically, we propose that the neuropeptides that may be used as indicators of substance use disorders and recovery are orexin (also known as hypocretin) and oxytocin. Neuropeptide transmitters are made exclusively in hypothalamic neurons and have extensive central nervous system projections. Harris & Aston-Jones [14] reveal a novel and important role for the orexin/hypocretin neuronal system in reward processing and addiction. The hypothalamus has been considered essential in motivational behaviors. Orexin is expressed in the lateral hypothalamus and has been implicated in the regulation of feeding behavior. Neurons expressing this neuropeptide have extensive projections to regions of the brain vital to behavioral responses to drugs of abuse, raising the possibility that these pathways are central when examining addiction [15,16].

Some neuropeptides affect adaptive central nervous system processes related to opiate, ethanol, and cocaine addiction. Oxytocin, a neuropeptide synthesized in the brain and released at the posterior pituitary, is also released in the central nervous system. Oxytocin acts within the CNS to inhibit the development of tolerance to morphine. The behavioral tolerance to the effect of cocaine was inhibited by oxytocin while increasing behavioral sensitivity to cocaine. Tolerance to ethanol was also inhibited by oxytocin [17].

A hormone that may be measured to indicate recovery is cortisol. It has been determined that addiction patients appear to be biologically different from healthy subjects as indicated by greater cortisol blunting and other cortisol-related hormonal and psychological responses [18]. In fact, during early drug-quitting phases, cortisol levels were found to drop and be related to withdrawal distress and could be found to be associated with the attainment of abstinence [19,20].

#### Chemical Dependency Domain

This domain represents the extent to which the individual uses addictive chemicals. It may be measured using the alcohol and drug use subscales of the ASI, and urine and breath screening. A five panel urine quick screen may be administered to test for benzodiazepines, amphetamines, cocaine, THC and opiates. If the result is positive a quantitative test may be conducted to test for ethanol, amphetamine, barbiturate, benzodiazepines, cannabinoids, cocaine (metabolite), opiates, phencyclidine, methadone, propoxyphene, meperidine, and tramadol. A breathalyzer may be utilized for alcohol testing.

#### Psychological Domain

Many persons suffering from addiction enter treatment in a significantly cognitively impaired state. Thus, improvement in gross cognitive functioning is indicative of recovery. Basic cognitive functioning may be measured with one of two instruments: the Folstein Mini-Mental State Examination (MMSE) [21] or the Test of Nonverbal Intelligence (TONI-3) [22]. The MMSE assesses orientation, registration, attention and calculation, recall, and language. Alternatively, the TONI-3 is administered to persons who have a cognitive impairment or who speak a language other than English. The test requires no reading, writing, speaking, or listening by the participant. Cognitive impairment in individuals with normal intelligence can be assessed using the Shipley Institute of Living Scale [23].

#### Psychiatric Domain

This domain refers to the presence of a mental disorder. This may be measured by a diagnosis on Axis I of the *Diagnostic and Statistical Manual of Mental Disorders *[3]. Recovery would be indicated by a diagnosis of a disorder in remission. This domain may also be measured by the psychiatric health subscale of the ASI, which captures a somewhat different aspect of psychiatric functioning than does the DSM diagnosis.

#### Family/Social Domain

This domain includes interaction with family and engagement with community. It may be measured using the employment/self support, family relations, and illegal activity subscales of the ASI, and the Work Interference, Family Problems, Antisocial Practices Scales of the Minnesota Multiphasic Personality Inventory-2 (MMPI-2) [24].

#### Spiritual Domain

This domain reflects the wide usage of Alcoholics Anonymous and other 12-step programs in the addiction rehabilitation community. It is defined as how an individual's way of life is reflected in thinking, speaking, and acting and the quality of one's relationships with oneself, others, and with a Higher Power. Spirituality may be measured with the Spirituality Self-Rating Scale [25] and the Spirituality Scale of the ASI-JCAHO Version [26].

### Testing the Model

The next step in this research process is to test the hypothesized conceptual model. Such a test would involve collecting data using all of the proposed instruments at intake into a treatment program and at predetermined time points thereafter. Following data collection, confirmatory factor analysis would be used to examine (1) the extent to which the proposed measurable indicators are representative of their respective conceptual domains, (2) the extent to which changes in the indicators occur over time; and (3) the extent to which the conceptual domains are correlated with each other initially and over time.

Confirmatory factor analysis would yield both an estimation of how well the model fit the data, and tests of specific hypotheses. For purpose (1) above, the null hypothesis is that each of the indicators within a domain are not correlated with each other. If so, this would demonstrate an absence of an underlying concept. However, if the null hypothesis were rejected, it would suggest that there is construct validity of the underlying concept or domain. For purpose (2), the null hypothesis would be that the indicators do not change over time as individuals proceed through the treatment and aftercare process. Rejection of the null hypothesis would suggest that these indicators are in fact indicative of recovery. For purpose (3), the null hypothesis is that the conceptual domains are not correlated; rejection would suggest convergent validity of the model. Additionally, this model could be further validated by correlating propensity scores derived from the model with clinicians' independent ratings of the clients' degree of recovery (i.e., known-groups validity).

As a result of this validation process, it is likely that the model would be refined. Some of the indicators may turn out not to adequately represent their respective domains (i.e., indicators not correlated with their domains), and some of the domains may turn out not to adequately represent recovery (i.e., domains not correlated with other domains). Thus, the model would likely be reduced through empirical testing. Individual components of the domains and biomarkers would need to be checked for sensitivity and specificity which would address effect size issues.

## Discussion and Conclusions

This study has several limitations that should be noted. First, the participants represented a narrow selection of experts. Second, the study site, the Hanley Center, serves a fairly demographically homogeneous treatment population, which might bias the experts' views. Third, some of the conceptual domains have more support in the treatment literature than others; for example, the biomarker domain primarily has research but not treatment support; however the aim of the proposed model is to test the treatment support for this domain.

This conceptual model represents an initial effort to build upon and expand upon traditional biopsychosocial models of addiction such as the syndrome model proposed by Schaffer et al. [27]. The syndrome model proposes a common etiology (e.g., distal antecedents such as neurobiology and proximal antecedents such as biopsychosocial events) that creates multiple expressions of the disease of addiction such as drug and alcohol misuse, gambling, smoking, psychiatric co-morbidities, social misconduct and biological malfunctioning. The syndrome model describes addiction. This paper proposes a potential model of recovery by incorporating elements of the syndrome model and reversing this process to build a model of recovery. The recovery model proposes that the reversal of addiction can be captured biologically through the measurement of neuroadaptations and hormonal shifts, and psychosocially with paper and pencil measures across the chemical dependency, psychiatric, psychological, family/social, and spiritual domains.

Additionally, the proposed model expands upon traditional biopsychosocial models by adding the bio-marker domain and the spiritual domain. In testing the bio-marker domain, neuropeptides and neuroadaptations may be examined to identify certain genes, DNA and core RNA for biological elements that are diagnostic of addiction, representative of recovery, and predictive of relapse. Additionally, cortisol may be examined to see if there are relationships between this hormone, stress, addiction, and recovery. Adding the spiritual domain incorporates the work of Galanter [8] who looks at addiction and recovery by examining how spiritual interventions can be used to support recovery efforts.

If the hypothesized conceptual model is supported by empirical data, it could provide a starting point for the development of a comprehensive, unifying perspective of recovery and its measurable indicators that could potentially be used to assess recovery in future outcome studies. This model would yield a conception of recovery as a continuum, rather than as a dichotomy (i.e., a person is either in recovery or not). In this model, the greater the improvement over time on a greater number of indicators, the greater the "recovery" would be considered to be. Additionally, a validated unifying model could provide a common metric across treatment programs that could allow for direct comparisons of effectiveness.

## Competing interests

The authors declare that they have no competing interests.

## Authors' contributions

KD designed the study, supervised the literature review, led the panel discussions, and drafted the manuscript. BK participated in the panel discussions and contributed the conceptualization of the physical domain. PJK contributed the conceptualization of the bio-marker domain. All authors read and approved the final manuscript.

## Authors' information

KD is the Director of Research at the Hanley Center, West Palm Beach, Florida. Before joining Hanley Center, from 2005 through 2009, KD was the Senior Health Planner for the Palm Beach County Health Department, where she was the lead in Mobilizing for Action through Planning and Partnerships (MAPP) for Palm Beach County, Florida. KD provided epidemiological reports on one-hundred and seventy health indices for the county and compared these findings with the State and Nation. These reports are considered "Best Practices" by the Florida State Department of Health. Starting in 2004 KD was an Assistant Professor at the University of Miami: Miller School of Medicine; Department of Epidemiology and Public Health, and is currently the Director, Comprehensive Substance Abuse and Mental Health Research Center at Hanley (under the University of Miami's auspices. From 2006-present KD was and is a Clinical Professor in the College of Osteopathic Medicine at NOVA Southeastern University. For over twenty years, Dr. Dodge has built expertise in the chemical dependency industry in substance abuse, mental health, community health and sexually transmitted infections. Dr. Dodge is a quantitative and qualitative epidemiological consultant, has published in numerous peer-reviewed journals and has conducted and published pioneering studies in specific substance abuse areas.

BK is Chief Executive Officer and Medical Director of Research of the Hanley Center. She has been practicing medicine for over thirty years. She is American Board of Addiction Medicine certified as well as board certified in Family Practice. Prior to joining Hanley Center in 2001, BK was the Medical Director for the Center for Alcohol and Drug Studies. From 1987 to 1999, she was the Medical Director at Cornerstone, a substance abuse treatment program at Wellington Regional Medical Center, and maintained a private family practice that included pediatrics and geriatrics. BK served as Clinical Assistant Professor of Behavioral Medicine at Nova Southeastern University in North Miami Beach from 1994 to 2000. She also taught addiction medicine to 1st and 2nd year students at the University of Miami Miller School of Medicine on the FAU Campus. In 1999 she developed the Inner City Outreach Program and the Medical Mobile Unit of West Palm Beach, the first such project undertaken in Palm Beach County. BK is a member of the American Medical Association, the American Medical Women's Association, the American College of Family Practitioners, the American Osteopathic Association and the American Society of Addiction Medicine. She has lectured on numerous topics, including the bio-chemistry of the brain as it relates to addiction. In 2008 the Palm Beach County Medical Society honored BK as a Heroes in Medicine award recipient for her leadership, active community philanthropy and commitment to the field of medicine. BK received a Bachelor of Science degree and was class valedictorian at Manhattanville College in Purchase, New York. At the University of Miami (Florida) she graduated Phi Kappa Phi with a Master of Science degree. After studying at Des Moines University, Health Sciences, she served an internship in Osteopathic Medicine in North Miami Beach. She completed her family practice residency at Miami's Jackson Memorial Hospital. In 2007 she was certified by the Medical Review Officer Certification Council (MTOCC) as a Medical Review Officer. Certified MROs evaluate drug and alcohol test results in the workplace.

PJK is Associate Professor in the Department of Molecular Therapeutics at Scripps Florida. His research focuses on understanding the neurobiological mechanisms underlying psychiatric disorders such as drug addiction, depression and anxiety, with a particular emphasis on the role of nicotinic acetylcholine receptors (nAChRs) in these processes. He employs a multidisciplinary approach that includes complex behavioral paradigms, physiological analyses, and molecular biological techniques. Current projects include the utilization of vector-based delivery of short-interfering RNAs (siRNA) into the brains of rodents to identify novel signaling cascades that may play a role in addiction-like behaviors. PJK received a B.A. in Biochemistry from Trinity College Dublin, Ireland, and a Ph.D. in Neuropsychopharmacology from King's College London, UK. He has received the Post-Doctoral Fellowship Award (2001-2003) from The Peter F. McManus Charitable Trust; the Young Scientist Travel Award (2001) from the International Society for Neurochemistry; the Early Career Investigator Award (2004) from the National Institute on Drug Abuse; the Young Investigator Travel Award (2004) from the American College of Neuropsychopharmacology; and the Young Investigator Award (2004-2006) from the National Alliance for Research on Schizophrenia and Depression (NARSAD).
